# Prediction of Mineral Composition in Commercial Extruded Dry Dog Food by Near-Infrared Reflectance Spectroscopy

**DOI:** 10.3390/ani9090640

**Published:** 2019-09-01

**Authors:** Arianna Goi, Carmen L. Manuelian, Sarah Currò, Massimo De Marchi

**Affiliations:** Department of Agronomy, Food, Natural Resources, Animals and Environment, University of Padova, Viale dell’Università 16, 35020 Legnaro (PD), Italy

**Keywords:** dog, NIR, dog nutrition, extruded pet food

## Abstract

**Simple Summary:**

Mineral content in dog food is essential to ensure animals’ adequate development and health status, but its analysis is time-consuming and companies are not always equipped with the technology to perform it. Near-infrared spectroscopy (NIRS) is a rapid, objective, easy to manage, chemical-free, and non-destructive method that is already available in the food industry for the prediction of gross composition (e.g., moisture, protein, fat, etc.). However, this technological approach is not yet used for the prediction of minerals because there is scarce information regarding the feasibility of NIRS to predict minerals in pet food. Results of this study revealed that, among all minerals analyzed, adequate NIRS prediction models were obtained for S and K for extruded dry dog food. The development of prediction models for mineral content in dry dog food opens the possibility of on-line and at-line analyses of minerals in the products during the manufacturing process, which could help the manufacturing decision support system in the pet food industry.

**Abstract:**

The pet food industry is interested in performing fast analyses to control the nutritional quality of their products. This study assessed the feasibility of near-infrared spectroscopy to predict mineral content in extruded dry dog food. Mineral content in commercial dry dog food samples (*n* = 119) was quantified by inductively coupled plasma optical emission spectrometry and reflectance spectra (850–2500 nm) captured with FOSS NIRS DS2500 spectrometer. Calibration models were built using modified partial least square regression and leave-one-out cross-validation. The best prediction models were obtained for S (coefficient of determination*; R^2^* = 0.89), K (*R^2^* = 0.85), and Li (*R^2^* = 0.74), followed by P, B, and Sr (*R^2^* = 0.72 each). Only prediction models for S and K were adequate for screening purposes. This study supports that minerals are difficult to determine with NIRS if they are not associated with organic molecules.

## 1. Introduction

Companion animals’ population is increasing worldwide, which has contributed to the growth of the pet food industry over the last decade in terms of sold volume and revenues [1]. The constant request for a better quality product from the animals’ owners makes the pet food sector particularly careful in ensuring foods that are nutritionally adequate for the health and welfare of the animals. Complete dog food has to supply the nutrients necessary to fulfil companion animals’ daily nutritional requirements without impairing animals’ health. Minerals play an important role in animals’ health and development. Calcium, P, and Mg are associated with the development and maintenance of bones and teeth, as well as muscle development. Potassium and Na are responsible for the body’s acid-base and fluid balance, and S is necessary for the dog’s hair, skin, and nails. Nevertheless, the excess of some minerals could impair animals’ health and be detrimental for the bioavailability of some trace minerals such as Fe [2,3] causing anemia, Mn [4,5] decreasing growth rate and leading to soft-tissue calcification, Cu [6,7] contributing to the development of hepatic and neurological disorders, and Zn [8,9,10] affecting skin, hair, and animals’ growth. Boron and Sr are related to Ca metabolism [11]. Molybdenum intervenes in several enzymatic reactions, an excess of Cr is related to gastroenteritis, dermatitis, kidney insufficiency, and liver damage, and Ni deficiency affects development and reproduction of the animal, as well as absorption and deposition of other minerals [12]. Vanadium has been demonstrated to have a hypoglycemic effect in dogs [13], and toxicity of Li [14], Ba [15], and Al [12] have been reported in dogs. On the other hand, the restriction of P intake has been recommended to prevent the appearance of kidney diseases [16,17,18], the reduction of Mg and P intake to diminish the probability to develop struvite uroliths [19], and the reduction of Ca and P intake to decrease the risk of calcium oxalate stones [20] and skeletal disorders [21]. To help owners in their buying decisions, it is mandatory for mineral content to be indicated on the label of companion animal food, depending on the ingredients used [22].

The analytical determination of minerals requires time and labor; it is expensive, and many pet food manufacturers may not have a laboratory equipped with the necessary instruments to perform such analyses. Therefore, the pet food industry is interested in more cost-effective and fast methods for the determination of minerals. Near-infrared spectroscopy (NIRS) is a rapid, objective, easy to manage, chemical-free, and non-destructive method that provides analysis of several traits simultaneously at a lower cost than the common reference analyses [23,24]. This technology is routinely used in most pet food factories for the determination of gross composition of their products, such as moisture, protein, and fat. It is difficult to achieve accurate prediction models for the determination of minerals with infrared spectroscopy because they do not have a specific absorption band in the infrared region [25]. The bonds that could be predicted with NIRS are O–H, C–H, N–H, and S–H [26], because NIRS responds to the bonding energies of hydrogen [27]. However, minerals can be predicted if they are part of organic complexes [27], or due to the alteration that minerals produce in the water region of the spectrum [25,27,28]. Prediction models for some minerals have been successfully proposed for cheese (Ca, P, S, Mg, Zn, and Cu [29]; Na and K [30]; Ca and Zn [31]) and processed meat products (Na [32]; [33,34]). Although, only one study has evaluated NIRS ability to determine minerals in pet food, showing unsatisfactory results in dry dog food [35]. Thus, the aim of the present study was to assess the feasibility of near-infrared spectroscopy to predict mineral content in extruded dry dog food.

## 2. Materials and Methods

### 2.1. Sample Selection

A total of 119 dry extruded commercial dog food sealed packages of 2, 2.5, and 3 kg were collected from September 2017 to January 2019 from a pet food factory in North Italy (Dorado S.r.l., Monsole di Cona, Venice, Italy) and stored in the dark at room temperature until their analysis. Samples were analyzed at the food laboratory of the Department of Agronomy, Food, Natural resources, Animals and Environment of the University of Padova (Legnaro, Italy) after receiving the product. A description of the dog food varieties included in this study based on the main protein source is displayed in Table 1 and gross composition (moisture, crude protein, ether extract, crude fiber, ash, and nitrogen-free extract) in Table 2. The different pet food compositions and the number of samples for each main protein source represent the availability of these products in the Italian pet food market. Moreover, pet food varieties included were intended for puppy and adult dogs, and small, medium, and large dog breed sizes. To develop more accurate NIRS prediction models, it is important to have a large variability of the reference data [36] and a set of samples that represents the population that will be analyzed in the future [35]. According to a standardized internal laboratory protocol, 100 g of each package was ground with a knife mill (Retsch Grindomix GM200, Retsch GmbH & Co, Haan, Germany) to pass through a 1 mm screen, and divided into two aliquots; the first one for the reference analysis and the second one for the NIRS analysis.

### 2.2. Mineral Reference Analyses

Major mineral (Ca, P, Mg, Na, K, and S) and trace mineral (Al, B, Ba, Cr, Cu, Fe, Li, Mn, Mo, Ni, Sr, V, and Zn) analyses were performed by mineralization of 350 mg of each ground sample in closed vessels with nitric acid in a microwave digestion system (Ethos 1600 Milestone S.r.l., Sorisole, Bergamo, Italy). Samples were diluted in ultrapure water to obtain a final volume of 25 mL, and then concentrations were quantified with inductively coupled plasma optical emission spectrometry (ICP-OES) Ciros Vision EOP (Spectro Analytical Instruments GmbH, Kleve, Germany). The ICP-OES determined Ca at 317.933 nm, P at 178.287 nm, Mg at 285.213 nm, Na at 589.592 nm, K at 766.941 nm, S at 182.034 nm, Al at 167.078 nm, B at 249.677 nm, Ba at 455.404 nm, Cr at 267.716 nm, Cu at 324.754 nm, Fe at 259.941 nm, Li at 670.780 nm, Mn at 257.611 nm, Mo at 202.095 nm, Ni at 231.604 nm, Sr at 407.771 nm, V at 292.464 nm, and Zn at 213.856 nm. All the minerals quantified were above the limit of detection of the instrument (0.001 ppm), with the exception of 11 samples for V.

All instrument operating parameters were optimized for acid solution and the conditions of ICP-OES were 2 mL/min of sample aspiration rate, plasma power 1350 W, coolant flow 11 L/min, auxiliary flow 0.60 L/min, nebulizer flow 0.75 L/min, and integration time of 28 s. Calibration standards for each mineral were prepared from monoelement solutions (Inorganic Ventures, Christiansburg, VA, USA) with 5% nitric acid and 65% Suprapur at concentration of 0, 1, 2, 5, 10, 20, 50, and 100 mg/L.

### 2.3. Near-Infrared Spectra Collection

To obtain the spectra of each sample, 50 g of ground product was placed in a large sample FOSS cup (diameter 105 mm, depth 35 mm; FOSS Electric A/S, Hillerød, Denmark) and scanned with NIRS DS2500 (FOSS Electric A/S, Hillerød, Denmark) every 0.5 nm from 850 to 2500 nm wavelength at room temperature. Each spectrum was an average of 32 sub-spectra recorded at eight different points by rotating the sample FOSS cup automatically. Spectra were collected through ISIscan Nova and Mosaic software (FOSS Electric A/S, Hillerød, Denmark) and recorded as log(1/reflectance). 

### 2.4. Chemometric Data Analysis

The chemometric analysis was carried out using WinISI 4 software (Infrasoft International, Port Matilda, PA, USA). Modified partial least squares regression analysis was used to develop the prediction models for each mineral using leave-one-out cross-validation, which excludes a single sample in each iteration. In order to optimize the calibration accuracy for each mineral, i.e., to obtain a higher coefficient of determination and residual predictive deviation, three passes of outlier elimination were applied. A critical *T*-value of 2.5 was set for *T* outliers, thus samples for which the predicted value differed more than 2.5 standard error of cross-validation from the reference one were removed. Several combinations of scatter corrections (NONE, no correction; D, detrending; SNV, standard normal variate; SNV + D, standard normal variate and detrending; MSC, multiplicative scatter correction; WMSC, weighted multiplicative scatter correction; ISC, inverted scatter correction) and mathematical treatments [0,0,1,1; 1,4,4,1; 1,8,8,1; 2,5,5,1; 2,10,10,1; where the first digit is the number of the derivative, the second is the gap over which the derivative is calculated, the third is the number of data points in the first smoothing, and the fourth is the number of data points in the second smoothing] were tested [37].

The best model for each mineral was selected based on the number of latent factors (LF), the coefficient of determination of calibration (R^2^C) and of cross-validation (R^2^CrV), the standard error of calibration (SEC) and of cross-validation (SECrV), and the residual predictive deviation of cross-validation (RPD), which was calculated as the ratio between SD and SECrV [38]. Interpretation of R^2^CrV was based on Karoui et al. [39], who reported that a prediction model with a coefficient of determination between 0.66 and 0.81 could give an approximate quantitative estimation of the reference value, a coefficient of determination between 0.82 and 0.90 could give a good estimation, and above 0.91 could give an excellent estimation. Interpretation of RPD was based on Williams [40] for complex feed matrix, who indicated the applicability of the prediction models based on the RPD obtained. A prediction model with an RPD below 1.9 is not recommended to be used, an RPD between 2.5 and 2.9 could be applied for screening, an RPD above 3.0 could be adequate for quality control, and an RPD above 4.0 could be adequate for any application [40]. Moreover, normality of residuals of the predicted models was assessed and a *t*-test was performed to determine whether the bias differed statistically from zero using SAS ver. 9.4 (SAS Institute Inc., Cary, NC, USA).

## 3. Results

### 3.1. Chemical Composition

The most abundant mineral was Ca (13.57 g/kg in dry matter basis, DM), followed by P (9.94 g/kg DM) and K (7.19 g/kg DM; Table 3). The lowest variability among major minerals was observed for Mg (coefficient of variation (CV) = 17%) and the greatest one for K (CV = 40%), with a CV above 30% for Na, P, Ca, and S. A total of 13 trace minerals were quantified, being the most abundant Fe (379.87 mg/kg DM) and the lowest one Li (0.19 mg/kg DM; Table 3). The lowest variability among trace minerals was observed for Fe and Mn (CV = 25%), and the greatest one for Sr (CV = 61%), with a CV above 30% for the rest of minerals.

### 3.2. Near-Infrared Spectroscopy Prediction Models

**Figure 1:**
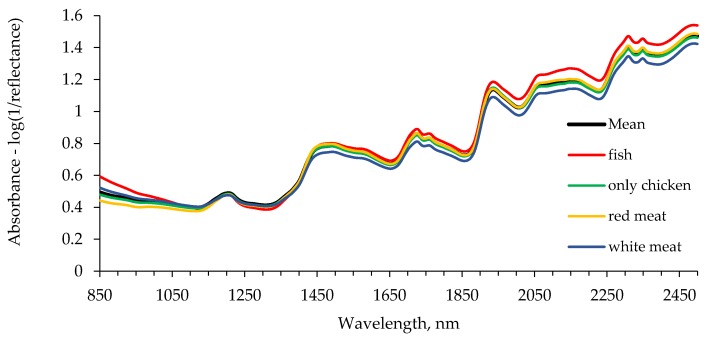
Average raw spectra of extruded dry dog food using near-infrared spectroscopy for the complete dataset (*n* = 119), and groups according to the main protein source: Red meat group (*n* = 37), fish group (*n* = 13), mixed group (*n* = 15), chicken group (*n* = 29), and white meat group (*n* = 21).

Average raw absorbance spectrum for extruded dry dog food is depicted in Figure 1. The average raw spectra, as well as the spectra of the samples considering the groups indicated in Table 1, followed the same pattern. On the raw spectrum, a first and defined peak was observed at 1208 nm and a wider peak that included wavelengths from 1450 to about 1505 nm. Further peaks were found close to 1726, 1760, and 1938 nm. Moreover, a wide peak from 2060 to 2170 nm, and two peaks at 2307 and 2350 nm were also observed.

The statistics of the best NIRS prediction models for each mineral are reported in Table 4. Outliers detected were <11% for all the minerals, except for Cu (14% of outliers). Latent factors ranged from 8 (Ca and Na) to 10 (P) for major minerals, and from 1 (Cu and Cr) to 10 (Fe) for trace minerals. The most selected scatter correction was D, and half of the equations chosen were developed applying a second derivate, over a gap of ten or five data points.

**Figure 2:**
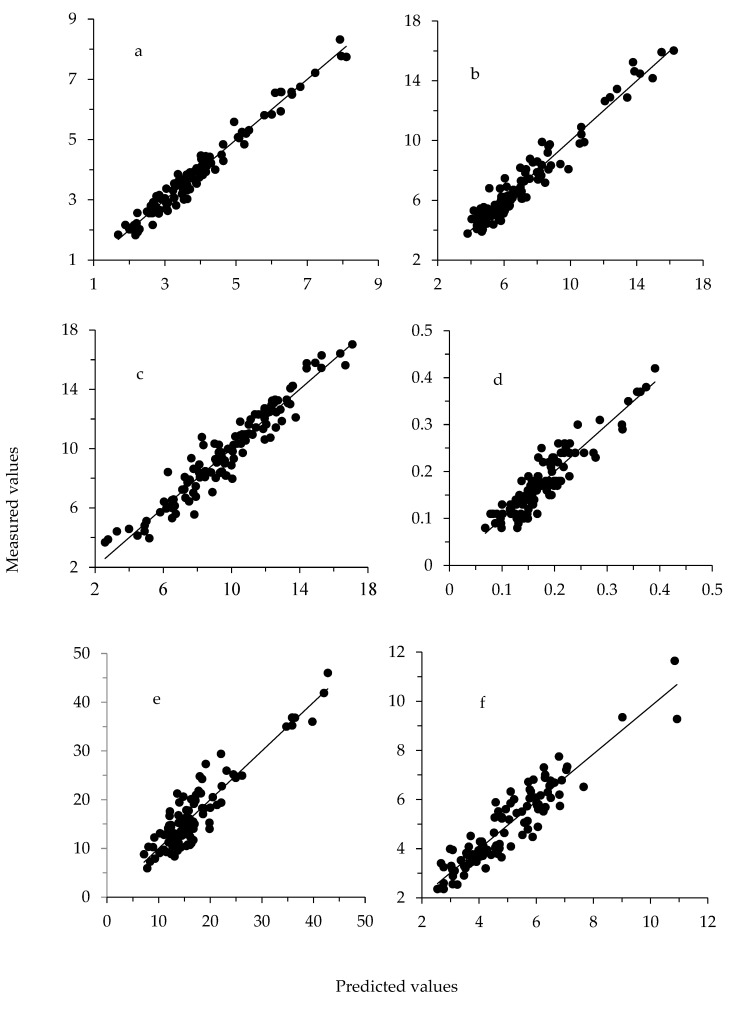
Linear regression plot of measured versus predicted values (dry matter basis, DM) for (**a**) S, g/kg DM; (**b**) K, g/kg DM; (**c**) P, g/kg DM; (**d**) Li, mg/kg DM; (**e**) Sr, mg/kg DM; and (**f**) B, mg/kg DM. Values in leave-one-out cross-validation (*n* = 119).

The R^2^C was greater than the R^2^CrV, and the best prediction models were obtained for S (RPD = 3.04) and K (RPD = 2.58). Moreover, the minerals with greater RPD were those with greater CV. For trace minerals, prediction equations with RPD close to 2.0 were obtained for Li, B, and Sr. Considering major minerals, the prediction model with the lowest accuracy was obtained for Ca (RPD = 1.49), whereas among trace minerals they were obtained for Cr, Mn, and Cu (RPD between 1.10 and 1.16). The linear regression of measured versus predicted values for the best three major and trace minerals prediction equations (R^2^CrV > 0.66) are represented in Figure 2. Residuals of those prediction equations were normally distributed and bias did not differ from zero.

## 4. Discussion

### 4.1. Chemical Composition

The average gross composition and minerals of the samples were consistent with FEDIAF recommendations [41]. The greater abundance of Ca, P, and K among all the major minerals agreed with several authors [35,42,43]. In contrast with our results, Alomar et al. [35] reported the lowest variability for K and the greatest for Mg and Ca, with a CV below 30% for P and Na; whereas Pereira et al. [43] observed the lowest CV for P and the greatest for Ca, with a variability that ranged from 27 to 38%. Moreover, Alomar et al. [35] and Pereira et al. [43] determined some trace minerals in dog food. Alomar et al. [35] reported similar values for Fe, Cu, Mn, and Zn, but a greater variability of Mn content. Pereira et al. [43] reported a similar quantity of Cu and Mn, but a lower content of Fe with greater variability, and a greater content of Zn with a similar variability compared with the present study.

### 4.2. Near-Infrared Spectroscopy Prediction Models

The average NIRS raw spectrum obtained followed a similar shape to those previously reported in unground [44] and ground dog food samples [35]. Peaks nearby 1208, 1728, 1762, 2308, and 2348 nm are assumed to be related to lipids. In particular, they have been assigned to a CH second overtone (1208 nm), CH first overtone (1728 and 1762 nm), and CH stretch and deformation in a CH_2_ group (2308 and 2348 nm; [31]). However, Hervera et al. [45] indicated that the peak at 1210 nm in pet food corresponded to absorption by OH groups in carbohydrates. Peaks at 1502, 2056, and 2174 nm have been associated to NH stretch, NH stretch and amide II, and amide I and amide III, respectively [31]. Peaks around 1452 and 1938 nm have been also related to water; to an OH stretch first overtone and an OH bend second overtone, respectively [31].

Although minerals do not have a specific absorption band in the infrared spectroscopy region, the ability of NIRS to predict some minerals has been related to the bonds of minerals to proteins or other organic molecules as reported in cheese [29], to the S-H bonds [26], and to the effect of the element on the water absorption band [25,27,28], such as for Na [32] and K [46,47]. However, it has been described that P forms only establish weak hydrogen bridges due to their low electronegativity making difficult its determination with NIRS [47].

Infrared spectroscopy capacity to determine mineral content has been evaluated on several food matrices [48] but, to the best of our knowledge, only Alomar et al. [35] have evaluated the feasibility of NIRS to predict minerals in pet food. The percentage of samples removed as outliers partially agreed with Manuelian et al. [29], who reported <14% for almost all major and trace minerals in cheese, and De Marchi et al. [32], who reported between 4 and 9% of outliers for Na in several groups of processed meat products. The most accurate calibrations were obtained mainly using the scatter correction D, similarly to what was reported for Na in meat products [32,46]. On the other hand, Alomar et al. [35] obtained better results not using a scatter correction. The most used mathematical treatments agreed to what was reported by Alomar et al. [35] in dog food and Manuelian et al. [29] in cheese. The high number of LF showed the difficulty in obtaining accurate prediction models for minerals from near-infrared spectrum information, which agreed with the results in cheese [29,49] and processed meat products [32].

Minerals that presented greater CV showed higher R^2^CrV, which supports that better prediction models may be related to a wider range of variability. Although it is commonly agreed that the higher the RPD the better the model, researchers do not agree on the interpretation of RPD at the low end of the range. Based on Williams’ interpretation of the RPD [40], the K prediction model could be adequate for screening purposes and the S prediction model could be adequate for quality control. Being S the best predicted mineral could be related to the capacity of NIRS to detect the S-H bonds [26], which are related to the sulfhydryl groups in proteins. All the other mineral prediction models were below 2.5, which is the threshold to consider an equation fair enough to be used [40]. Alomar et al. [35] did not achieve a good calibration model for any mineral, reporting RPD between 1.1 (Fe) and 1.8 (Cl and Mg). The highest RPD reported by Alomar et al. [35] was lower than the one obtained in our study. However, the RPD for Mg reported by those authors was greater than the one in the present study (RPD = 1.6), whereas we obtained greater RPD for Ca (1.5), P (1.9), Na (1.6), and K (2.6) than they did.

On the other hand, a greater accuracy of the prediction models to predict Ca (RPD = 4.57), Na (RPD = 3.28), P (RPD 4.02), S (RPD = 4.22), Mg (RPD = 3.71), and Zn (RPD = 3.9) has been reported in commercial cheeses [29] than in the present study. Also, predictions of Ca (R^2^CrV = 0.76), Na (R^2^CrV = 0.64), Fe (R^2^CrV = 0.84), and Zn (R^2^CrV = 0.70) in meat [46] and Na in processed meat products (RPD = 3.38 to 5.71) [32] were more accurate than the values reported in Table 4. The greater accuracy of the mineral prediction equations could be related to the matrix of study and the manufacturing process. Minerals that were better predicted in cheese and meat are those minerals that were probably linked to organic complexes or that interfered with the water of the product; for example, Ca, Mg, and P in cheese are part of the casein micelle, and a huge quantity of NaCl is used during the brining process of cheese [29] or processed meat products [32], increasing the level of Na in the product. As reviewed by De Marchi et al. [36], the absorbance depends on the number of molecular bonds; the lower the quantity of the element, the lower the number of molecular bonds that can be excited and detected by NIRS. Another aspect that negatively affects the feasibility of NIRS to predict minerals in compound food for animals is that minerals, and often trace minerals, are usually added to the product in inorganic form to adjust their amount to the animals’ requirements. As indicated above, the capacity of NIRS to detect minerals is based on the association of those elements to organic complexes [27,36], so the inorganic forms are less likely to be detected using NIRS [27]. However, the feasibility of NIRS to predict minerals from inorganic salts could be related to the association of the cations to organic or hydrated inorganic molecules [27]. Thus, the low accuracy of the prediction models obtained for minerals in dog food could be partially related to the low quantity of the elements in the product, especially when referring to trace minerals, and to the addition of inorganic salts during the manufacturing process.

## 5. Conclusions

In conclusion, results of the present study demonstrate that, among all the minerals analyzed, NIRS prediction models for S (R^2^CrV = 0.89; RPD = 3.04) and K (R^2^CrV = 0.85; RPD = 2.58) could be used for screening purposes in extruded dry dog food. This study supports the hypothesis that minerals are difficult to determine using NIRS if they are not associated with organic molecules or hydrated inorganic molecules. Based on these results, further studies with wet pet food could be interesting to confirm that hypothesis.

## Figures and Tables

**Table 1:** Classification by main protein source of the commercial extruded dry food samples for dogs included in the study.

Group	Main Protein Sources	Other Ingredients	Number of Samples
Red meat	Pork, lamb, horse, venison	Eggs, pea, rice, potato	37
Fish	Only fish	Potato, rice	13
Mixed	Chicken, pork, fish	-	15
Chicken	Only chicken	Rice	29
White meat	Rabbit, chicken, duck	Eggs, potato	21
Other	Not specified on the label	-	4

**Table 2:** Chemical composition (% as fed) of extruded dry dog food samples (*n* = 119) included in this study, determined using near-infrared spectroscopy, applying pre-installed commercial calibrations of FOSS for pet food.

Trait	Mean	SD	Minimum	Maximum
Moisture	5.56	1.22	2.50	8.00
Crude protein	28.14	3.95	21.26	43.09
Ether extract	13.71	2.20	8.12	17.86
Crude fiber	3.36	0.94	1.92	9.38
Ash	6.25	1.21	3.25	10.24
Nitrogen-free extract ^1^	42.98	5.91	23.70	54.05

SD = standard deviation; ^1^ Nitrogen-free extract = 100 – (moisture + crude protein + ether extract + crude fiber + ash).

**Table 3:** Mineral composition (dry matter basis, DM) of extruded dry dog food samples (*n* = 119) included in the present study quantified with inductively coupled plasma optical emission spectrometry.

Mineral	Mean	SD	Minimum	Maximum	CV
Major minerals, g/kg DM					
Ca	13.57	4.89	4.34	37.69	36.1
P	9.94	3.24	3.68	19.41	32.6
K	7.19	2.88	3.77	16.02	40.0
Na	5.34	1.64	1.23	9.66	30.8
S	3.87	1.43	1.54	8.32	37.0
Mg	1.21	0.21	0.80	1.97	17.3
Trace minerals, mg/kg DM					
Fe	370.87	90.89	128.58	702.70	24.5
Zn	190.24	57.07	37.77	357.53	30.0
Al	152.83	54.99	65.54	307.38	36.0
Mn	74.66	18.39	19.48	122.16	24.6
Cu	25.58	8.02	11.08	55.34	31.3
Sr	18.77	11.45	5.94	72.25	61.0
Ba	5.60	2.47	1.47	18.63	44.1
B	5.08	1.73	2.36	11.65	34.1
Cr	1.74	0.89	0.54	5.26	50.9
Ni	1.28	0.45	0.57	3.81	34.8
Mo	0.86	0.40	0.25	2.70	45.9
V^1^	0.44	0.22	0.16	1.18	49.8
Li	0.19	0.09	0.08	0.65	47.3

SD = standard deviation; CV = coefficient of variation, %; ^1^ The number of samples used for V was 108.

**Table 4:** Fitting statistics for modified partial least square regression models developed for major (g/kg in dry matter basis ) and trace mineral content (mg/kg in dry matter basis) in ground commercial extruded dry dog food samples ^1^.

Item	Outliers	n	Scatter Correction ^2^	Math Treatment ^3^	LF	Mean	SD	R^2^C	SEC	R^2^CrV	SECrV	RPD
Major minerals												
Ca	7	112	D	1881	8	13.32	4.34	0.68	2.47	0.55	2.91	1.49
P	5	114	D	210101	10	9.80	3.14	0.91	0.92	0.72	1.66	1.89
K	6	113	MSC	210101	9	7.03	2.74	0.94	0.69	0.85	1.06	2.58
Na	6	113	ISC	210101	8	5.31	1.60	0.83	0.66	0.60	1.00	1.59
S	5	114	D	2551	9	3.82	1.36	0.96	0.26	0.89	0.45	3.04
Mg	10	109	ISC	1441	9	1.19	0.17	0.78	0.08	0.63	0.11	1.64
Trace minerals												
Fe	7	112	WMSC	210101	10	363.84	78.24	0.89	25.89	0.59	49.67	1.58
Zn	9	110	NONE	1441	7	189.46	50.20	0.65	29.89	0.49	35.78	1.40
Al	5	114	SNV	210101	4	149.25	52.70	0.66	30.76	0.52	36.47	1.44
Mn	9	110	SNV	1881	4	74.22	14.97	0.38	11.75	0.20	13.29	1.13
Cu	17	102	WMSC	2551	1	23.72	3.95	0.34	3.22	0.25	3.41	1.16
Sr	10	109	SNV + D	1441	9	16.65	7.44	0.83	3.08	0.72	3.92	1.90
Ba	6	113	MSC	2551	5	5.34	1.93	0.71	1.04	0.47	1.39	1.38
B	9	110	ISC	2551	6	5.06	1.72	0.86	0.64	0.72	0.91	1.90
Cr	11	108	D	0011	1	1.53	0.54	0.23	0.48	0.16	0.49	1.10
Ni	6	113	WMSC	1881	6	1.25	0.36	0.64	0.21	0.44	0.27	1.35
Mo	13	106	NONE	1881	6	0.76	0.24	0.66	0.14	0.50	0.17	1.43
V	5	103	SNV + D	2551	3	0.43	0.21	0.68	0.12	0.53	0.14	1.46
Li	10	109	D	1441	9	0.18	0.07	0.84	0.03	0.74	0.03	1.98

^1^ LF = optimal number of model factors; n = number of samples; R^2^C = R^2^ for calibration; SD = standard deviation; SEC = SE of calibration; R^2^CrV = R^2^ for the cross-validation; SECrV = SE of cross-validation; RPD = ratio of performance to deviation. ^2^ D = detrend; MSC = multiplicative scatter correction; ISC = inverted scatter correction; WMSC = weighted multiplicative scatter correction; NONE = no correction; SNV = standard normal variate; SNV + D = standard normal variate and detrending. ^3^ Derivate order, gap (nm), first smoothing, and second smoothing intervals (nm).
